# Supplementary material for: Interneuron-specific gamma synchronization indexes cue uncertainty and prediction errors in lateral prefrontal and anterior cingulate cortex
Source: eLife. 2021 Jun 18;10:e69111. doi: 10.7554/eLife.69111 (PMC8248985; doi:10.7554/eLife.69111)
Supplement: Supplementary file 1. [file elife-69111-supp1.docx]

Supplementary File 1

Cohen’s d effect sizes for firing rate modulation of each of eight *e-types* during the trial epochs Feature-1, Feature-2, and Reward for lateral prefrontal cortex (PFC) and anterior cingulate cortex (ACC).

| Condition | B5 | B4 | B3 | B2 | B1 | N3 | N2 | N1 |
| --- | --- | --- | --- | --- | --- | --- | --- | --- |
| Color | 0.153 | -0.327 | 0.107 | 0.262 | 0.104 | -0.300 | -0.491 | -0.294 |
| Motion | -0.261 | -0.038 | -0.258 | 0.218 | 0.293 | 0.105 | 0.434 | 0.285 |
| Color - Motion | 0.209 | -0.239 | 0.191 | 0.136 | 0.135 | -0.342 | -0.549 | -0.393 |

Figure 5-supplement 1A-C, Feature 2. Cohen’s d effect size in PFC

| Condition | B5 | B4 | B3 | B2 | B1 | N3 | N2 | N1 |
| --- | --- | --- | --- | --- | --- | --- | --- | --- |
| Motion | 0.198 | -0.083 | 0.289 | -0.140 | -0.265 | -0.083 | 0.232 | 0.407 |
| Color | 0.246 | -0.079 | 0.396 | -0.117 | -0.263 | -0.186 | -0.340 | 0.526 |
| Motion - Color | -0.184 | -0.108 | -0.157 | -0.161 | 0.214 | 0.231 | 0.351 | -0.348 |

Figure 5-supplement 1A-C, Feature 1. Cohen’s d effect size in PFC

| Condition | B5 | B4 | B3 | B2 | B1 | N3 | N2 | N1 |
| --- | --- | --- | --- | --- | --- | --- | --- | --- |
| Color | -0.143 | -0.056 | -0.021 | 0.061 | 0.108 | 0.071 | 0.242 | -0.382 |
| Motion | -0.100 | -0.007 | -0.012 | 0.213 | 0.088 | -0.051 | -0.038 | -0.324 |
| Color - Motion | -0.054 | -0.031 | -0.010 | -0.105 | 0.026 | 0.106 | 0.254 | -0.025 |

Figure 5-supplement 1D-F, Feature 2. Cohen’s d effect size in ACC

| Condition | B5 | B4 | B3 | B2 | B1 | N3 | N2 | N1 |
| --- | --- | --- | --- | --- | --- | --- | --- | --- |
| Motion | 0.041 | -0.022 | 0.186 | -0.286 | 0.018 | 0.050 | 0.269 | -0.389 |
| Color | -0.045 | 0.051 | 0.213 | -0.255 | 0.119 | -0.032 | 0.143 | -0.497 |
| Motion -Color | 0.088 | -0.056 | -0.044 | -0.006 | -0.119 | 0.079 | 0.078 | 0.332 |

Figure 5-supplement 1D-F, Feature 1. Cohen’s d effect size in ACC

| Condition | B5 | B4 | B3 | B2 | B1 | N3 | N2 | N1 |
| --- | --- | --- | --- | --- | --- | --- | --- | --- |
| Reward | 0.171 | -0.031 | -0.172 | 0.061 | -0.311 | -0.045 | 0.302 | -0.016 |

Figure 5-supplement 1G, Reward. Cohen’s d effect size in PFC

| Condition | B5 | B4 | B3 | B2 | B1 | N3 | N2 | N1 |
| --- | --- | --- | --- | --- | --- | --- | --- | --- |
| Reward | 0.147 | -0.016 | -0.117 | -0.119 | 0.065 | 0.059 | -0.367 | 0.632 |

Figure 5-supplement 1H, Reward. Cohen’s d effect size in ACC
